# LazyB: fast and cheap genome assembly

**DOI:** 10.1186/s13015-021-00186-5

**Published:** 2021-06-01

**Authors:** Thomas Gatter, Sarah von Löhneysen, Jörg Fallmann, Polina Drozdova, Tom Hartmann, Peter F. Stadler

**Affiliations:** 1 Biology Department, Universidad Nacional de Colombia, Carrera 45 # 26-85, Edif. Uriel Gutiérrez, Bogotá, D.C Colombia; 2 Institute of Biology, Irkutsk State University, RU-664003 Irkutsk, Russia; 3 Bioinformatics Group, Department of Computer Science, and Interdisciplinary Center for Bioinformatics, Universität Leipzig, Härtelstraße 16–18, 04107 Leipzig, Germany; 4 Max Planck Institute for Mathematics in the Sciences, Inselstraße 22, 04103 Leipzig, Germany; 5 Department of Theoretical Chemistry, University of Vienna, Währinger Straße 17, 1090 Vienna, Austria; 6 Santa Fe Institute, 1399 Hyde Park Rd., Santa Fe, NM87501 USA

**Keywords:** Nanopore sequencing, Illumina sequencing, Genome assembly, Spanning tree, Unitigs, Anchors

## Abstract

**Background:**

Advances in genome sequencing over the last years have lead to a fundamental paradigm shift in the field. With steadily decreasing sequencing costs, genome projects are no longer limited by the cost of raw sequencing data, but rather by computational problems associated with genome assembly. There is an urgent demand for more efficient and and more accurate methods is particular with regard to the highly complex and often very large genomes of animals and plants. Most recently, “hybrid” methods that integrate short and long read data have been devised to address this need.

**Results:**

LazyB is such a hybrid genome assembler. It has been designed specificially with an emphasis on utilizing low-coverage short and long reads. LazyB starts from a bipartite overlap graph between long reads and restrictively filtered short-read unitigs. This graph is translated into a long-read overlap graph *G*. Instead of the more conventional approach of removing tips, bubbles, and other local features, LazyB stepwisely extracts subgraphs whose global properties approach a disjoint union of paths. First, a consistently oriented subgraph is extracted, which in a second step is reduced to a directed acyclic graph. In the next step, properties of proper interval graphs are used to extract contigs as maximum weight paths. These path are translated into genomic sequences only in the final step. A prototype implementation of LazyB, entirely written in python, not only yields significantly more accurate assemblies of the yeast and fruit fly genomes compared to state-of-the-art pipelines but also requires much less computational effort.

**Conclusions:**

LazyB is new low-cost genome assembler that copes well with large genomes and low coverage. It is based on a novel approach for reducing the overlap graph to a collection of paths, thus opening new avenues for future improvements.

**Availability:**

The LazyB prototype is available at https://github.com/TGatter/LazyB.

## Background

The assembly of genomic sequences from high throughput sequencing data has turned out to be a difficult computational problem in practice. Recent approaches combine cheap short-read data (typically using Illumina technology [1]) with long reads produced by PacBio or Nanopore technologies [2]. Although the short-read data are highly accurate and comparably cheap to produce, they are insufficient even at (very) high coverage due to repetitive elements. Long-read data, on the other hand, are comparably expensive and have much higher error rates. HiFi PacBio reads [3] derived from repeat sequencing of circularized elements rival short read accuracy but at vastly increased costs.

Several assembly techniques have been developed recently for de novo assembly of large genomes from high-coverage (50$\times$ or greater) PacBio or Nanopore reads. Recent state-of-the-art methods employ a hybrid assembly strategy using Illumina reads to correct errors in the longer PacBio reads prior to assembly. For instance, the 32 Gb axolotl genome was produced in this manner [4].

Traditional assembly strategies can be classified into two general categories [5, 6]. The Overlap-layout-consensus (OLC) assembly model attempts to find all pairwise matches between reads, using sequence similarity as a metric for overlaps. A general layout is constructed and post-processed in various ways. Most notably, overlaps can be transformed into assembly graphs such as string graphs [7]. This method is flexible to read length and can be adapted to the diverse error models of different sequencing technologies. However, finding all overlaps is very expensive, in particular as read-sizes become large.

In de Bruijn graph strategies [8, 9], reads are deconstructed to fixed length *k*-mers, representing nodes. Edges are inserted between nodes that overlap on $(k-1)$-mers. Ideally, a de Bruijn graph thus represents exactly one Eulerian path per chromosome, although this property is generally violated in practice due to sequencing errors even in the absence of repetitive elements. With the help of specialized hashing strategies, *k*-mers can be efficiently stored and constructed. Thus, de Bruijn graphs require much less memory than OLC strategies. An overall speed up can be attributed to the absence of an all-*vs*-all comparison step. However, as *k* has to be chosen smaller than read size, contiguity information is lost. With increasing error rates in reads, de Bruijn graphs tend to become less useful, as *k*-mers become also less accurate.

Long read only and hybrid assembly strategies also largely align to these two categories, although some more unique methods have emerged over the years. Canu [10] and Falcon [11] implement the classic OLC approach, albeit both error-correct long reads before creating a string graph. MinHash filters [12] can significantly reduce the costs of comparisons, but overall complexity remains high. Wtdbg2 [13] also follows OLC, but utilizes de Bruijn like graphs based on sparse *k*-mer mapping for comparison. It avoids all-*vs*-all mapping by matching reads that share *k*-mers under the assumption that even under high error rates correct pairs share more *k*-mer than those with spurious matches. Shasta [14] implements a full de Bruijn graph strategy by transforming *k*-mers into a run-length encoding that is more robust to sequencing errors in long reads. Newer versions of Canu also implement a similar encoding [15].

Classic de Bruijn methods have been adapted to combine both long and short reads into a hybrid assembly. Long reads can serve as “bridging elements” in the same way as mate pairs to resolve paths in (short read) assembly graphs [16, 17].

Under the assumption that short-read assemblies are cheap and reliable, various workflows have been proposed to integrate both kinds of data also for OLC-like approaches. As a general goal, these programs avoid the costly all-*vs*-all comparison to create the assembly graph with the help of various heuristics. MaSuRCA [18] attempts to join both long and short reads into longer super-reads by chaining unique *k*-mers, thereby reducing the number of reads that need to be tested for overlap. WENGAN [19] first creates full short-read contigs that are then scaffolded by synthetic mate pairs generated out of the long reads. Flye [20], even more uniquely, assembles intentionally erroneous contigs that are concatenated to a common sequence. Self-mapping then reveals repeats that can be resolved much like in a traditional assembly graph.

HASLR [21] defines an assembly-graph-like structure, that includes both short and long reads. Short reads are assembled into contigs that, after *k*-mer filtering to remove repeats, are aligned to long reads. In the resulting backbone graph, short-read contigs serve as nodes that are connected by an edge if they map onto the same long read. While different to e.g. string graphs, standard tip and bubble removal algorithms are applied to remove noise. Contigs are extracted as paths. TULIP [22] implements a very similar strategy, however, does not assemble short reads into full contigs. Instead, the gaps between mate-pairs are closed if possible with sufficiently rare *k*-mers, resulting in relative short but unique seeds that serve in the same capacity. In both cases, consensus construction of the resulting sequence is trivial. Edges define fixed regions on groups of long reads that can be locally aligned for each edge along a path.

DBG2OLC [23] is methodologically most closely related to LazyB. The two approaches, however, differ in several key features. DBG2OLC assembles short reads to full contigs, thereby avoiding repeat resolving techniques such as gap closing or scaffolding because these introduce too many errors. The short-read Contigs are then aligned against the long reads. Each long read implies a neighborhood of contigs. Mappings are corrected prior to graph construction via consistency checks over all neighborhoods for each contig, i.e., contigs are required to map in the same order on all long reads. This technique can help to remove both spuriously matched contigs and chimeric long reads, but requires sufficient coverage to allow for effective voting. Similar to LazyB, long reads serve as nodes in the DBG2OLC graph, with edges representing contigs mapping to two long reads. Nodes that map a subset of contigs of another node are removed as they are redundant. The resulting graph is error corrected by classic tip and bubble removal, after which paths are extracted as contigs, following the edge with the best overlap at each step. LazyB instead uses a step-wise procedure to extract paths that employs a series of heuristics to edit the initial overlap graph to a collection of paths.

LazyB implements an alternative approach to assembling genomes from a combination of long-read and short-read data. It not only avoids the expensive, direct all-*vs*-all comparison of the error-prone long-read data, but also the difficult mapping of individual short reads against the long reads, and the conventional techniques to error-correct de Bruijn or string graphs. As we shall see, its step-wise approach of processing the long-read overlap graph adds the benefit of producing quite good assemblies with surprisingly low requirements on the coverage of both short and long reads. Even at just 2× coverage of long reads the contiguity of assemblies of complex genomes can be significantly improved using our method. It lends itself in particular to the exploratory assembly of a large numbers of species, where cheap identification of functional islands is required rather then expensive finishing.

This contribution, which is a revised, updated, and extended version of a paper presented at WABI 2020 [24], is organized as follows. We first outline the overall strategy of LazyB and then describe the pre-processing of the short-read data and the mapping short-reads-derived unitigs to long reads. Similar to DBG2OLC and HASLR, LazyB operates on a long-read overlap graph whose edges are derived from partially assembled short-read sequences that map to multiple long-reads. The main innovation in $\texttt {LazyB}$ is the *Processing of the overlap graph*, which proceeds by a series of heuristics inspired by properties of overlap graphs derived from ideal data, and avoids the commonly used techniques to correct assembly graphs. We then briefly describe the construction of the sequence assembly from the path decomposition of the overlap graph. Benchmarking results are reported for the assembly of yeast, fruitfly, and human genomes. We close with a discussion and an outlook to open problems and future improvements.

## Strategy

**Fig. 1:**
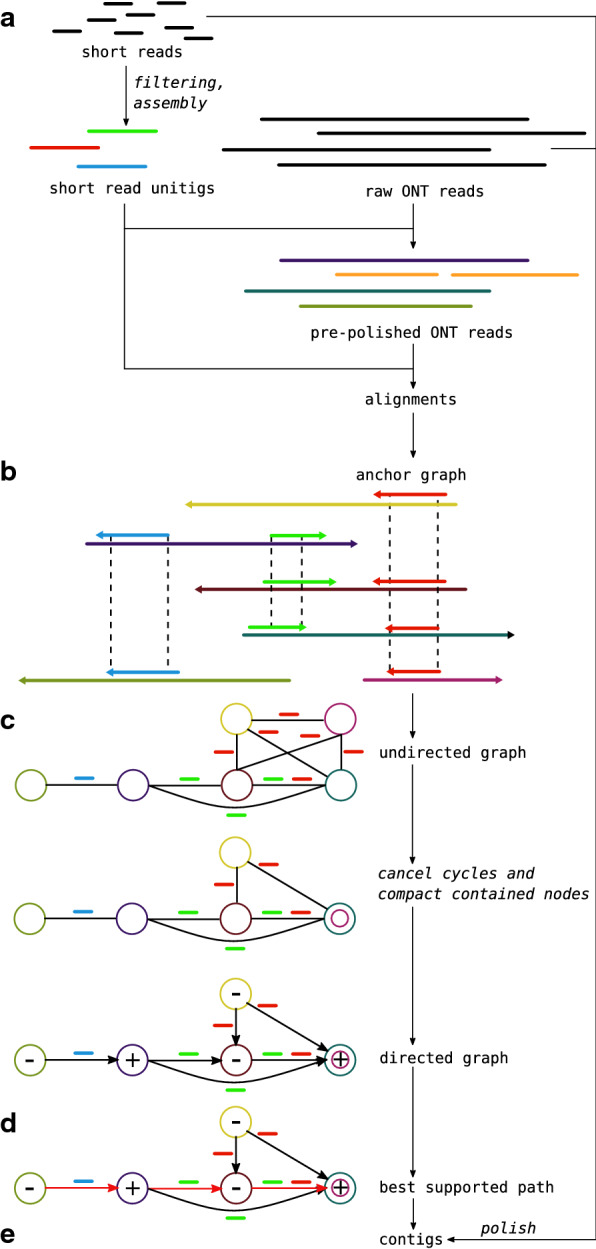
Overview of the LazyB assembly pipeline. (**a**) Short Illumina reads are filtered to represent only near unique *k*-mers and subsequently assembled into unambiguous unitigs. Long Nanopore reads (ONT) can be optionally scrubbed to include only regions consistent to at least one other read. For larger data sets scrubbing can be handled on subsets efficiently. Mapping unitigs against Nanopore reads yields unique “anchors” between them (**b**). An undirected graph (**c**) is created by adding Nanopore reads as nodes and edges between all pairs of reads sharing an “anchor”. Each edge is assigned a *relative orientation*, depending on whether the “anchor” maps in the same direction on both Nanopore reads. Cycles with a contradiction in orientation have to be removed before choosing a node at random and directing the graph based on its orientation. As Nanopore reads that are fully contained within another do not yield additional data, they can be collapsed. Contigs are extracted as maximally supported paths for each connected component (**d**). Support in this context is defined by the number of consistent overlaps transitive to each edge. Final contigs (**e**) can be optionally polished using established tools

LazyB does not pursue a “total data” approach. Instead, it identifies “anchors” that are nearly guaranteed to be correct and implements an, overall, greedy-like workflow to obtain very large long-read contigs. To this end, the initial overlap graph is first oriented and then edited in several consecutive steps to graph classes that more closely approach the desired final results, i.e., a union of paths. Conceptually, therefore, LazyB does not attempt solve a single global optimization problem but instead approximates as sequence of graph editing problems. This strategy of LazyB is outlined in Fig. 1.

The key idea to obtain the overlap graph is to start from a collection ${\mathcal {S}}:=\{s_i\}$ of pre-assembled, high-quality sequences that are unique in the genome. These are obtained from accurate short-read sequencing data and serve as “anchors” to determine overlaps among the long reads ${\mathcal {R}}:=\{r_j\}$. In practice, ${\mathcal {S}}$ can be obtained by assembling Illumina data with low or moderate coverage to the level of unitigs only. The total genomic coverage of ${\mathcal {S}}$ only needs to be large enough to provide anchors between overlapping long reads. This data is therefore rigorously filtered to be devoid of repetitive and highly similar sequences. Mapping a sequence $s\in {\mathcal {S}}$ against the set ${\mathcal {R}}$ of long reads implies (candidate) overlaps $r_1-r_2$ between two long reads (as well as their relative orientation) whenever $s\in {\mathcal {S}}$ maps to both $r_1$ and $r_2$. Thus we obtain a directed overlap graph *G* of the long reads without an all-vs-all comparison of the long reads.

A series of linear-time filtering and reduction algorithms then prunes first the underlying undirected overlap graph and then the directed version of the reduced graph. Its connected components are reduced to near-optimal directed acyclic graphs (DAGs) from which contigs are extracted as best-supported paths. In the following sections the individual steps will be described in detail. In comparison to DBG2OLC we avoid global corrections of short-read mappings, but instead rely on the accuracy of assembled unitigs and a series of local corrections. For this, we utilize previously unreported properties of the class of alignment graphs used by both tools. This allows LazyB to operate reliably even on very low coverage. Variations of the dataset dependent assembly options have little impact on the outcome. In contrast to the complicated setup of options required for tools such as DBG2OLC, LazyB comes with robust defaults.

## Data preprocessing

A known complication of both PacBio and Nanopore technologies are chimeric reads formed by the artificial joining of disconnected parts of the genome [25] that may cause mis-assemblies [26]. Current methods dealing with this issue heavily rely on raw coverage [27] and hence are of little use for our goal of a low-coverage assembler. In addition, start- and end-regions of reads are known to be particularly error-prone [28]. We pre-filter low quality regions, but only consider otherwise problematic reads later at the level of the overlap graph.

### Short-read unitig-level assembly

Short-read (Illumina) data is preprocessed by adapter clipping and trimming. The set ${\mathcal {S}}$ of high quality fragments is obtained from a restricted assembly of the short-read data. The conventional use case of assembly pipelines aims to find a minimal set of contigs in trade-off to both correctness and completeness. For our purposes, however, completeness is of less importance and fragmented contigs are not detrimental to our workflow, as long as their lengths stay above a statistical threshold. Instead, correctness and uniqueness are crucial. We therefore employ two initial filtering steps:

(1) Using a *k*-mer profile, we remove all *k*-mers that are much more abundant than the expected coverage since these are likely part of repetitive sequences. This process can be fully automated.

(2) In order to avoid ambiguities, only branch-free paths are extracted from the short-read assembly graph. This feature is implemented e.g. in the de Bruijn graph assembler ABySS [29], which allows to assemble up to unitig stage. Moreover, a minimal path length is required for a unitig to serve as a secure anchor.

**Fig. 2:**
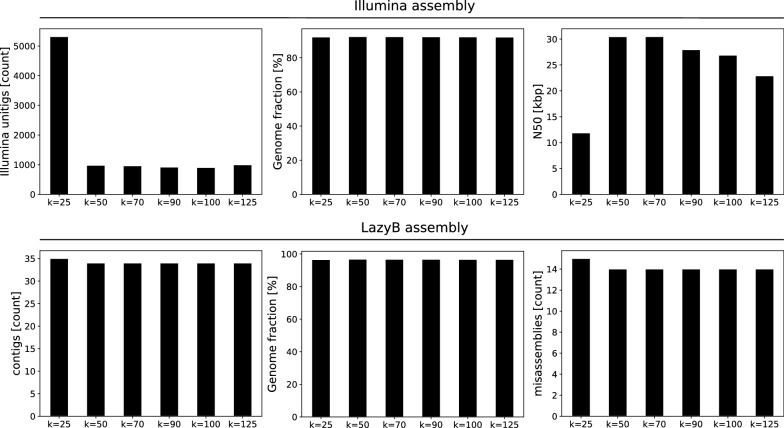
Assembly statistics as a function of the *k*-mer size used to construct unitigs from the short-read data for yeast. Top: Illumina unitigs (left: number of unitigs; middle: fraction of the reference genome covered; right: N50 values); bottom: final LazyB assembly at ${\sim}11\times$ long reads (left: number of unitigs; middle: fraction of the reference genome covered; right: number of mis-assemblies)

Since repeats in general lead to branch-points in the de Bruijn graph, repetitive sequences are strongly depleted in unitigs. While in theory, every such assembly requires a fine-tuned *k*-mer size, a well known factor to be influential on assembly quality, we found overall results to be mostly invariant of this parameter. To test this, we systematically varied the *k*-mer-size for ABySS. Nevertheless, we found little to no effect on the results of LazyB (Fig. 2). As assembly stops at unitigs, error rates and genome coverage stay within a narrow range as long as the unitigs are long enough.

**Fig. 3:**
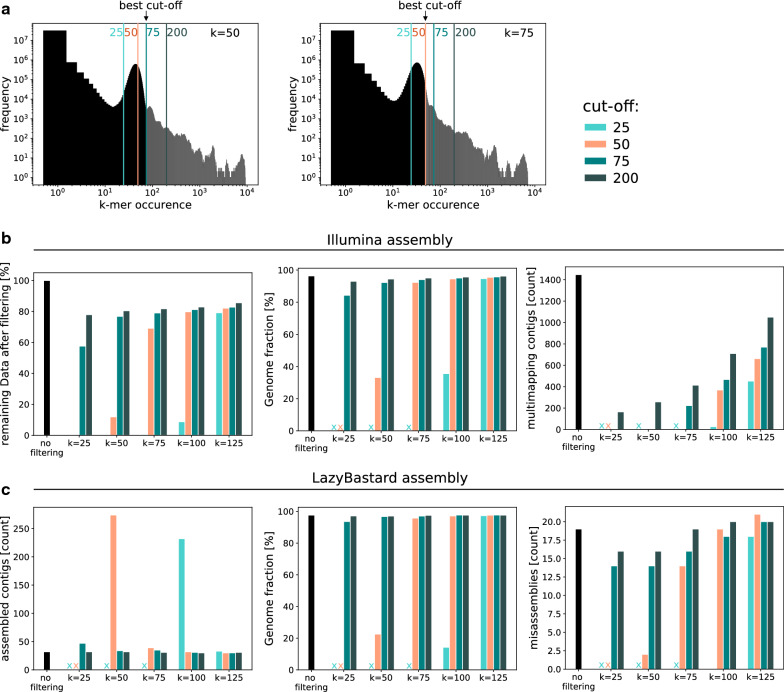
Assembly statistics of yeast as a function of the *k*-mer size and maximal occurrence cut-off used to remove very frequent *k*-mers from short reads prior to unitig assembly. (**a**) *k*-mer profiles for *k* = 50 bp and *k* = 75 bp. Cut-offs restrict short reads to different degrees. Note logarithmic axes. (**b**) Illumina unitigs (left: percentage of remaining short-read data; middle: fraction of the reference genome covered; right: number of unitigs mapping multiple times to reference). (**c**) Final LazyB assembly left: number of unitigs; middle: fraction of the reference genome covered; right: number of mis-assemblies). x: not enough data to assemble

**Fig. 4:**
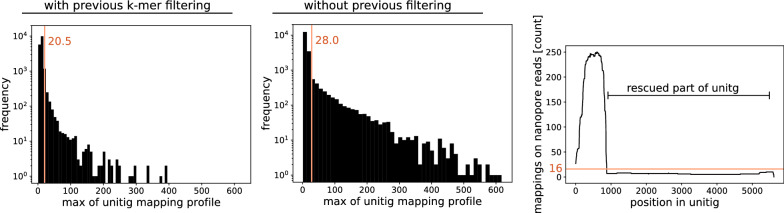
Exclusion of unitigs based on very high mapping coverage. Thresholds are IQR × 1.5+Q3. Shown are maximal values of coverage profiles for unitigs assembled with (left) and without (middle) previous *k*-mer filtering. Note the logarithmic axes. right: exemplary profile; only the high-coverage peak is excluded. Threshold is Q3

The strategies for filtering short-read data have a larger impact than the choice of the *k*-mer size for unitig assembly (Fig. 3). This is not surprising given that both chimeric unitigs and unitigs that harbor repetitive DNA elements introduce spurious edges into the long-read overlap graph *G* and thus negatively influence the assembly. In order to exclude short reads that contain highly frequent *k*-mers, the maximal tolerated occurrence has to be set manually and is dependent on the *k*-mer size. Setting the cut-off right next to the main peak in the profiles has turned out to be a good estimate. After assembling short reads, unitigs are mapped to long reads and a coverage profile over the length of every unitig is calculated. Unitigs with maximal coverage above interquartile range $IQR\times 1.5+Q3$ are considered outliers. However, regions below coverage threshold (Q3) spanning more than 500 bp can be “rescued”. This filter step effectively reduces ambiguous regions, in particular when no previous filtering has been applied (Fig. 4). Combining both short-read filters improves the assembly quality; see Table 1.

**Table 1:** Impact of short-read filtering strategies on LazyB assembly quality in fruit fly

Filter strategy	Compl. [%]	#ctg	#MA
no filter	82.81	457	302
*k*-mer filter	80.66	567	104
unitig filter	80.71	563	108
*k*-mer and unitig filter	80.11	596	99

Column descriptions: *compl*eteness of the assembly, #ctg: number of contigs, #MA: number of mis-assemblies (breakpoints relative to the reference assembly)

### Anchor alignments

The set ${\mathcal {R}}$ of long reads is mapped against the unitig set. At present we use minimap2 [30] for this purpose. Regions or whole unitigs significantly exceeding the expected coverage are removed from ${\mathcal {S}}$, as described in the last section, because they most likely are repetitive or at least belong to families of very similar sequences such as coding sequences of multi-gene families. Note that all repetitive elements connected to a unique region within a single long read may still be correctly assembled.

**Fig. 5:**
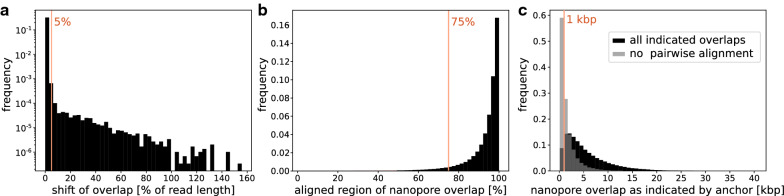
Consistency test of anchor-linked long-read overlaps to direct alignments of both reads on fruit fly. (**a**) Frequencies of shifted offsets (% of the longer read); changes up to 5% are tolerated; note logarithmic axis. (**b**) Frequencies of the percentage at which the direct alignment covers the overlap. A minimum of 75% is set for consistency. (**c**) Long read pairs where no direct alignment is possible tend to have shorter anchor-indicated overlaps. Connections that cannot be confirmed via direct alignments despite an expected overlap of at least 1 kbp are excluded

Classic alignment tools perform poorly in the presence of high rates of insertions and deletions (InDels) [31]. Even methods specifically advertised for this purpose rely on scoring schemes that cannot accurately represent the extreme abundance of InDels in long-read data. Instead, they rely on seeds of high quality matches that are then chained with high error tolerance. Currently, minimap2 [30, 32] is one of the most commonly used tools for this purpose. Since we do not have a gold standard set of perfect data, we can only roughly estimate the influence of this heuristic on the LazyB alignment quality in a related experiment. Specifically, we tested consistency of anchor alignments on pairs of long reads to direct alignments of both reads for fruit fly. Consistency is validated at the level of relative orientation, the offset indicated by both alignment methods, the portion of overlap that can be directly aligned and whether direct alignment of the long reads is possible at all. Different relative orientations were observed only in very small numbers. Changes in the offset by more then 5% of the longer read length are equally rare (Fig. 5).

However, requiring a direct alignment of at least 75% of the overlap region marks 4.6% of the anchor links as incorrect. Removing these “incorrect” anchors, surprisingly, has a negative effect on the final LazyB assembly and in particular tends to break correct contigs apart; see Table 2. In our test set 7.7% of direct alignments of two anchor-linked long reads gave no result. In these cases, expected overlaps are rather short (Fig. 5). We therefore tested whether the assembly could be improved by excluding those connections between long reads for which no alignment could be calculated despite the presence of an overlap of at least 1 kbp (3.7%). We found, however, that this procedure also causes the loss of correct edges in *G*.

**Table 2:** Assessment of different parameters to verify long-read overlaps and their impact on LazyB assembly quality on fruit fly

Varification parameters	Compl.[%]	#ctg	#MA
Direction	80.13	608	111
Direction + offset	80.08	622	103
Direction + offset + incomplete mapping	80.04	1263	121
No mapping	80.15	801	113

Overlaps are indicated by anchors and evaluated by pairwise long-read alignments. They are considered valid if: the relative direction suggested by the anchor matches that of the pairwise alignment (direction); the offset is sufficiently similar for both methods (offset); at least 75% of the overlap is found as direct alignment (incomplete mapping); the overlap indicated by the anchor is less than or equal to 1 kbp or a pairwise alignment is possible (no mapping). Column descriptions: *compl*eteness of the assembly, #ctg: number of contigs, #MA: number of mis-assemblies (breakpoints relative to the reference assembly)

Summarizing, we observe three facts: (1) The overwhelming number of pairs is consistent and therefore true. (2) Removing inconsistent edges from the assembly not only does not improve the results but results are worse on average. (3) While we can manually identify some incorrect unitig matches, the mappings produced by minimap2 are too inconsistent for proper testing. Since we have no proper methods to identify such false positives we also cannot properly estimate the number of false negatives, i.e., missing matches in the graph , e.g. by computing a transitive completion.

Overall, out tests indicate that a high level trust in the anchors mapping in warranted. We also conclude that minimap2 is sufficient for our purposes. However, the data also suggest that the assembly would profit substantially from a more accurate handling of the overlap alignments. This remains a problem for future research at this point.

### Long read overlap graph

As a result of mapping the short-read unitig to the long reads, we obtain a set of *significant matches* ${\mathscr {V}}:=\{(s,r) \in {\mathcal {S}} \times {\mathcal {R}} \mid \delta (s,r)\ge \delta _* \}$ with matching scores $\delta (s,r)$ that exceed a user-defined threshold $\delta _*$. The *long-read overlap graph* *G* has the vertex set ${\mathcal {R}}$. Conceptually, two long reads overlap, i.e., there should be an undirected edge $r_1r_2\in E(G)$ if and only if there is an $s\in {\mathcal {S}}$ such that $(s,r_1)\in {\mathscr {V}}$ and $(s,r_2)\in {\mathscr {V}}$. The choice of $\delta _*$ therefore has an immediate effect on the resulting graph. Setting $\delta _*$ low will allow more false-postive edges to be introduced into the graph, as spurious matches become more likely. Higher values of $\delta _*$ improve the confidence of matches but may remove true edges. In the current prototype, we set the matching score as the number of exactly aligned basepairs in the match and require at least 500 such exact basepairs. With increasing accuracy of long read basecallers, long read mappers, unitig assembly, and possibly also dependent on the organism, this value is subject to change. In practice, we employ an overall more restrictive but robust procedure to introduce edges in order to reduce the danger of introducing false-positive edges into *G*, mitigating also effects of slightly sub-optimal choices of $\delta _*$.

For two distinct long reads $r_1,r_2 \in {\mathcal {R}}$ with a common $s\in {\mathcal {S}}$, i.e., $(s,r_1), (s,r_2) \in {\mathscr {V}}$, we denote by [*i*, *j*] and [*k*, *l*], respectively, the sequence intervals on *s* that match intervals on $r_1$ and $r_2$. The intersection $[i,j]\cap [k,l]$ is the interval $[\max \{i,k\},\min \{j,l\}]$ if $k\le j$ and the empty interval otherwise. A non-empty intersection $[i,j]\cap [k,l]$ corresponds to a direct match of $r_1$ and $r_2$. The expected bit score for the overlap is estimated as

1 $$\begin{aligned} \omega (s,r_1,r_2):=&\frac{1}{2}(\min \{j,l\}-\max \{i,k\}+1) \\&\times \left( \frac{\delta (s,r_1)}{(j-i+1)} + \frac{\delta (s,r_2)}{(l-k+1)}\right) \end{aligned}$$

if $[i,j]\cap [k,l]\ne \emptyset$. For $[i,j]\cap [k,l]=\emptyset$ we set $\omega (s,r_1,r_2):=0$. For a given edge $r_1r_2\in E(G)$ there may be multiple significant matches, mediated by a set of unitigs ${\mathscr {S}}_{r_1r_2} := \{ s\in {\mathcal {S}} \mid (s,r_1), (s,r_2) \in {\mathscr {V}} \}$. In ideal data all these matches are co-linear and consistent with respect their orientation. In real data, however, this may not be the case. It is necessary, therefore, to handle inconsistencies.

For each significant match $(s,r)\in {\mathcal {V}}$ we define the *relative orientation* $\theta (s,r)\in \{+1,-1\}$ of the reading directions of the short-read scaffold *s* relative to the long read *r*. The relative reading direction of the two long reads (as suggested by *s*) is thus $\theta _s(r_1, r_2) = \theta (s,r_1)\cdot \theta (s,r_2)$.

The position of a significant match (*s*, *r*) defined on the unitig *s* on interval [*i*, *j*] corresponds to an interval $[i',j']$ on the long read *r* that is determined by the alignment of *s* to *r*. Due to the large number of randomly distributed InDels in the Nanopore data, the usual dynamic programming alignment strategies fail to produce accurate alignments. This is also the case for minimap2 [30], our preliminary choice, as it only chains short, high quality matches into larger intervals. Although more accurate alignments would of course improve the local error rate of the final assembled sequence, we expect very little impact on the overall assembly structure of the assembly from local details of the sequence alignments at (*s*, *r*) matches. We therefore record only the matching intervals and use a coordinate transformation $\tau _r$ that estimates the position $\tau _r(h)\in [i',j']$ for some $h\in [i,j]$ by linear interpolation:

2 $$\begin{aligned} \tau _r(h):= {\left\{ \begin{array}{ll} j' - (j - h) \frac{j' - i' + 1}{j - i + 1} &{} \text {if } j - h \le h - i;\\ i' + (h - i) \frac{j' - i' + 1}{j - i + 1} &{} \text {if } j - h > h - i.\\ \end{array}\right. } \end{aligned}$$

The values of $\tau _r(h)$ are rounded to integers and used to determine intersections of matches. We write $[i,j]_r := [\tau _r(i),\tau _r(j)]$ for the interval on *r* corresponding to an interval [*i*, *j*] of *s*.

#### Definition 1

Two unitigs $s,s'$ in ${\mathscr {S}}_{r_1r_2}$ are *consistent* if (i) $\theta _s(r_1,r_2)=\theta _{s'}(r_1,r_2)$, (ii) the relative order of $[i^{s},j^{s}]_{r_1}$, $[k^{s'},l^{s'}]_{r_1}$ on $r_1$ and $[i^{s},j^{s}]_{r_2}$, $[k^{s'},l^{s'}]_{r_2}$ on $r_2$ is the same.

For distinct long reads $r_1,r_2 \in {\mathcal {R}}$, Definition 1 enables us to determine $m\ge 1$ subsets ${\mathscr {S}}^1_{r_1r_2},...,{\mathscr {S}}^m_{r_1r_2}$ of ${\mathscr {S}}_{r_1r_2}$ such that each is maximal with respect to inclusion and contains only unitigs that are pairwise consistent with respect to $r_1$ and $r_2$. In addition, we may require that the difference between the distances of consecutive corresponding intervals on $r_1$ and $r_2$, respectively, is sufficiently similar. Computing the set ${\mathscr {S}}\in \{{\mathscr {S}}^1_{r_1r_2},...,{\mathscr {S}}^m_{r_1r_2}\}$ that maximizes the total bit score $\sum _{s\in {\mathscr {S}}}\omega (s,r_1,r_2)$ amounts to a classical chaining problem. It can can be solved by dynamic programming [33] in quadratic time w.r.t. the number $|{\mathscr {S}}_{r_1r_2}|$ of unitig-mediated matches. An edge $r_1r_2$ is inserted into *G* if the optimal total bit score $\Omega (r_1,r_2):= \sum _{s\in {\mathscr {S}}}\omega (s,r_1,r_2)$ exceeds a user-defined threshold. The *signature* $\theta (r_1,r_2)$ of the edge $r_1r_2\in E(G)$ is the common value $\theta _s(r_1,r_2)$ for all $s\in {\mathscr {S}}$.

**Fig. 6:**
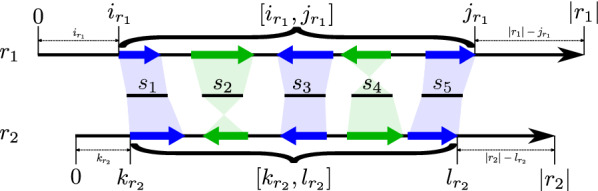
Construction of the overlap of two long reads $r_1$ and $r_2$ (long black arrows) from all unitigs ${\mathscr {S}}_{r_1r_2}:=\{s_1,...,s_5\}$ (short black bars) that match to both $r_1$ and $r_2$. A significant match (*s*, *r*) of $s\in {\mathscr {S}}_{r_1r_2}$ on $r\in \{r_1,r_2\}$ is illustrated by blue and green thick arrows on *r*. The relative orientation of (*s*, *r*) is indicated by the direction of its arrow, that is, $\theta (s,r)=+1$ (resp. $\theta (s,r)=-1$) if its arrow points to the right (resp. left). The subsets ${\mathscr {S}}^1_{r_1r_2}:=\{s_1,s_3,s_5\}$ (unitigs with blue significant matches) and ${\mathscr {S}}^2_{r_1r_2}:=\{s_2,s_4\}$ (unitigs with green significant matches) of ${\mathscr {S}}_{r_1r_2}$ are both inclusion-maximal and consists of pairwise consistent unitigs. The set ${\mathscr {S}}^1_{r_1r_2}$ maximizes $\Omega (r_1,r_2)$ and thus determines the overlap. It implies $\theta (r_1,r_2)=+1$. Moreover, $i_{r_1}$ (resp. $j_{r_1}$) is the minimal (resp. maximal) coordinate of significant matches of unitigs from ${\mathscr {S}}^1_{r_1r_2}$ on $r_1$. The corresponding coordinates on $r_2$ are $k_{r_2}$ and $l_{r_2}$, respectively. The spanning intervals $[i_{r_1},j_{r_1}]$ and $[k_{r_2},l_{r_2}]$ define the overlap of $r_1$ and $r_2$. In this example we have $i_{r_1}>k_{r_2}$ and $|r_1|-j_{r_1}>|r_2|-l_{r_2}$, implying that $r_2$ extends $r_1$ neither to the left or right and thus, edge $r_1r_2$ is contracted in *G*

For each edge $r_1r_2\in E(G)$ we furthermore determine $s,s' \in {\mathscr {S}}$ such that $\tau _{r_1}(i^s)$ is the minimal and $\tau _{r_1}(j^{s'})$ is the maximal coordinate of the matching intervals on $r_1$. Hence, the interval $[i^s,j^{s'}]_{r_1}$ spans all matching intervals on $r_1$. The corresponding pair of coordinates, $\tau _{r_2}(k^s)$ and $\tau _{r_2}(l^{s'})$, spans the matching intervals on $r_2$. In particular, the interval $[k^s,l^{s'}]_{r_2}$ (resp. $[l^{s'},k^s]_{r_2}$) spans both matching intervals on $r_2$ if $\theta (r_1,r_2)= 1$ (resp. $\theta (r_1,r_2)= -1$). For the sake of a clear notation, let $[i_{r_1},j_{r_1}]:=[i^s,j^{s'}]_{r_1}$ and $[k_{r_2},l_{r_2}]$ be the “spanning” interval on $r_2$, i.e., either $[k_{r_2},l_{r_2}]:=[k^s,l^{s'}]_{r_2}$ or $[k_{r_2},l_{r_2}]:=[l^{s'},k^s]_{r_2}$. Intervals $[i_{r_1},j_{r_1}]$ and $[k_{r_2},l_{r_2}]$ specify the known overlapping regions between $r_1$ and $r_2$, see also Fig. 6 for an illustration. If $\theta (r_1,r_2)=+1$ then $r_1$ *extends* $r_2$ *to the left* if $i_{r_1}>k_{r_2}$ and *to the right* if $|r_1|-j_{r_1}>|r_2|-l_{r_2}$. For $\theta (r_1,r_2)=-1$ the corresponding conditions are $i_{r_1}>|r_2|-k_{r_2}$ and $|r_1|-j_{r_1}>l_{r_2}$, respectively. If $r_1$ does not extend $r_2$ to either side then $r_1$ is completely contained in $r_2$ and does not contribute to the assembly. Similarly, if $r_1$ extends $r_2$ on both sides, $r_2$ is fully contained in $r_1$. In both cases we contract the edge between $r_1$ and $r_2$ in *G*. Otherwise, if $r_1$ extends $r_2$ to the left and $r_2$ extends $r_1$ to the right we record $r_1\rightarrow r_2$ and analogously, we set $r_1 \leftarrow r_2$ if $r_2$ extends $r_1$ to the left and $r_1$ extends $r_2$ to the right.

The result of this construction is a long-read-overlap graph *G* whose vertices are the non-redundant long reads and whose edges $r_1r_2$ record (1) the relative orientation $\theta (r_1,r_2)$, (2) the bit score $\Omega (r_1,r_2)$, (3) the local direction of extension, and (4) the overlapping interval.

## Processing of the overlap graph

### Consistent orientation of long reads

For perfect data it is possible to consistently determine the reading direction of each read relative to the genome from which it derives. This is not necessarily the case in real-life data. The relative orientation of two reads is implicitly determined by the relative orientation of overlapping reads, i.e., by the signature $\theta (r_1,r_2)$ of the edge $r_1r_2\in E(G)$. To formalize this idea we consider a subset $D\subseteq E(G)$ and define the *orientation* of *D* as $\theta (D):=\prod _{r_1r_2\in D} \theta (r_1,r_2)$. For a disjoint union of two edge sets *D* and $D'$ we therefore have  and, more generally, their symmetric different $D\oplus D'$ satisfies $\theta (D\oplus D')=\theta (D)\theta (D')$ since the edges in $D\cap D'$ appear twice in $\theta (D)\theta (D')$ and thus each of these edges contributes a factor $(\pm 1)^2=1$.

#### Definition 2

Two vertices $r_1,r_2\in V(G)$ are *orientable* if $\theta (P)=\theta (P')$ holds for any two paths *P* and $P'$ connecting $r_1$ and $r_2$ in *G*. We say that *G* is *orientable* if all pairs of vertices in *G* are orientable.

#### Lemma 3

*G* *is orientable if and only if every cycle* *C* *in* *G* *satisfies* $\theta (C)=1$.

#### *Proof*

Let $r,r'$ be two vertices of *G* and write ${\mathscr {C}}(r,r')$ for the set of all cycles that contain *r* and $r'$. If $r=r'$ or ${\mathscr {C}}(r,r')=\emptyset$, then *r* and $r'$ are orientable by definition. Now assume $r\ne r'$, ${\mathscr {C}}(r,r')\ne \emptyset$, and consider a cycle $C\in {\mathscr {C}}(r,r')$. Clearly, *C* can be split into two edge-disjoint path $C_1$ and $C_2$ both of which connect *r* and $r'$. If *r* and $r'$ are orientable, then $\theta (C_1)=\theta (C_2)$ and thus $\theta (C)=\theta (C_1)\theta (C_2)=1$. If *r* and $r'$ are not orientable, then there is a pair of paths $P_1$ and $P_2$ connecting *r* and $r'$ such that $\theta (P_1)=-\theta (P_2)$. Since  is an edge-disjoint union of cycles $C_i$ we have $-1 = \theta (P_1)\theta (P_2)=\prod _{i=1}^{k} \theta (C_i)$ and thus there is least one cycle $C_i$ with $\theta (C_i)=-1$ in *G*. $\square$

The practical importance of Lemma 3 is the implication that only a small set of cycles needs to be considered, because every cycle in *G* can be obtained as an $\oplus$-sum of cycles in a cycle basis [34, 35]. Every graph *G* with *c* connected components has a cycle basis comprising $|E|-|V|-c$ cycles. Particular cycles bases, known as *Kirchhoff bases*, are obtained from a spanning tree *T* of *G* as the set ${\mathscr {B}}$ of cycles $C_e$ consisting of the edge $e\in E\setminus T$ and the unique path in *T* connecting the endpoints of *e* [36]. Every cycle *C* of *G* can then be written as $C = \bigoplus _{e\in C\setminus T} C_e$, see e.g. [35].

#### Theorem 4

*Let* *G* *be a graph with signature* $\theta :E(G)\rightarrow \{-1,1\}$ *on its edges, and let* ${\mathscr {B}}$ *be a cycle basis of* *G*. *Then* *G* *is orientable if and only if* $\theta (C)=1$ *for all* $C\in {\mathscr {B}}$.

#### *Proof*

The theorem follows from Lemma 3 and the fact that every cycle *C* in *G* can be written as an $\oplus$-sum of basis cycles, i.e., $\theta (C)=1$ for every cycle in *C* if and only if $\theta (C')=1$ for every basis cycle $C'\in {\mathscr {B}}$. $\square$

Thm. 4 suggests the following, conservative heuristic to extract an orientable subgraph from *G*:

(1): Construct a maximum weight spanning tree $T_G$ of *G* by using the $\Omega$-scores as edge weights. Tree $T_G$ can easily be obtained using, e.g., Kruskal’s algorithm [37].
(2): Construct a Kirchhoff cycle basis ${\mathscr {B}}$ from $T_G$.
(3): For every cycle $C\in {\mathscr {B}}$, check whether $\theta (C)= -1$. If so, find the $\Omega$-minimum weighted edge ${\hat{e}} \in C$ and remove it from *E*(*G*) and (possibly) from $T_G$ if ${\hat{e}}\in E(T_G)$.

We delete the offending edge because it is very unlikely that the preprocessing correctly identified *that* two long reads overlap but failed to determine the correct relative orientation. The edge deletion is simplified by the following observation:

#### Lemma 5

*If* *T* *is maximal* $\Omega$-*weight spanning tree of* *T* *and end* *e* *is a non-tree edge, then* $\Omega (e)=\min _{e'\in C_e} \Omega (e')$.

#### *Proof*

Let $e'\in C_e\setminus \{e\}$ by a tree edge in the cycle $C_e$. Then $T'=T\setminus \{e'\}\cup \{e\}$ is again a spanning tree of *G* since the vertex set $V(C_e)$ is still connected and $T'$ contains not cycle. Its weight it weight is $\Omega (T')=\Omega (T)-\Omega (e')+\Omega (e)\le \Omega (T)$, since *T* is a maximum weight spanning tree by assumption. Thus $\Omega (e)\le \Omega (e')$, i.e., *e* has minimum $\Omega$-weight. $\square$

As a consequence, the minimum weight edge of an offending cycle is always the non-tree edge. Step (3) above therefore reduces to finding the basis edges ${\hat{e}}$ with negative signature cycles $C_{{\hat{e}}}$ and to remove these edges. The removal of ${\hat{e}}$ leaves $T_G$ unchanged and thus does not affect the contiguity of the assembly. The end result of the procedure outlined above is therefore a connected subgraph $G'$ and a spanning forest $T_{G'}=T_G$ for $G'$.

#### Lemma 6

*Let* *G* *be an undirected connected graph with signature* $\theta$ *and let* $G'$ *be the residual graph produced by the heuristic steps (1)-(3). Then (i)* $G'$ *is connected, (ii)* $G'$ *is orientable, and (iii)* $T_G$ *is an* $\Omega$-*maximal spanning tree of* $G'$.

#### *Proof*

(i) By Lemma 5, $T_G\subseteq E(G')$, hence $T_G$ is a spanning tree of $G'$ and thus $G'$ is connected. (ii) Since the heuristic removes all non-tree edges *e* with $\theta (C_e)=-1$, Thm. 4 implies that $G'$ is orientable. (iii) Since $T_G\subseteq E(G')$, Kruskal’s maximum weight spanning tree algorithm will pick the same spanning tree edges again from $E(G')$, and $T_G$ is an $\Omega$-maximal spanning tree. $\square$

In order to expedite the identification of edges that violate orientability in *G*, we define an orientation $\varphi$ for the vertices of *G*, i.e., the long reads. To this end, we pick an arbitrary $r_{*}\in V(G)$ as reference and set $\varphi (r_{*}):=+1$. Denote by $P_T(r)$ the unique path from $r^*$ to *r* and define $\varphi (r) := \theta (P_T(r))$.

#### Lemma 7

*If* *G* *is a connected orientable graph with signature* $\theta$, *then the vertex orientation* $\varphi$ *with reference* $\varphi (r_{*}):=+1$ *is independent of the choice of the spanning tree* *T*.

#### *Proof*

Let *P* be an arbitrary path connecting *r* and $r^*$. By connectedness, such a path exists and since *G* is orientable w.r.t. $\theta$ we have $\theta (P)=\theta (P_T)$. Furthermore *r* and $r^*$ are connected by the backbone of any spanning tree of *T*, $\varphi$ is independent of the choice of *T*. $\square$

As an immediate consequence we observe:

#### Corollary 8

*If* *G* *is an orientable graph with signature* $\theta$ *and vertex orientation* $\varphi$, *then every pair of adjacent vertices satisfies* $\varphi (r')\varphi (r'')=\theta (r'r'')$.

It follows that the heuristic to extract an orientable subgraph can be implemented in linear time:

(1): An $\Omega$-maximal spanning tree $T_G$ is obtained in ${\mathcal {O}}(|V|+|E|)$ time using Kruskal’s algorithm.
(2): The vertex orientation $\varphi$ is computed by traversal of the spanning tree $T_G$ in ${\mathcal {O}}(|V|)$ time.
(3): For each $e\in E\setminus T_G$, one checks in constant time whether $\varphi (r')\varphi (r'')\ne \theta (r'r'')$ and if so deletes the edge $r'r''$. The total effort is therefore ${\mathcal {O}}(|E|)$.

We remark that one could now define a graph ${\tilde{G}}$, obtained from *G* by inverting all long-reads *r* with a negative orientation $\varphi (r)=-1$. This amounts to replacing each long read *r* by its reverse complement. Since processing of the overlap graph does not explicitly consider the sequence information, it would be sufficient to replace the coordinates [*p*, *q*] of a match interval by $[\ell -q+1,\ell -p+1]$ and to invert the directionality of extension by another long read. The bit scores of matches, of course, remain unchanged. In ${\tilde{G}}$ all edge signatures are ${{\tilde{\theta }}}(e)=+1$. It is not necessary, however, to construct ${\tilde{G}}$ explicitly. Instead, we simply keep track of the vertex orientations $\varphi (r)$.

From here on, we again write *G* for the orientable graph $G'$.

### Reduction to a directed acyclic graph

We next make use of the direction of extension of long read $r_1$ and $r_2$ defined by the mutual overhangs in the case that $r_1r_2$ is an edge in *G*. We write  for the directed version of a connected component *G* of the residual graph $G'$ constructed above. For each edge $r_1r_2\in E(G)$ we create the corresponding edge $e \in E$( ) as

3 $$\begin{aligned} e:= {\left\{ \begin{array}{ll} r_1r_2 &{} \text {if }\varphi (r_1 ) = +1 \text { and } r_1 \rightarrow r_2 \text { or }\\ &{} \varphi (r_1 ) = -1 \text { and } r_1 \leftarrow r_2; \\ r_2r_1 &{} \text {if }\varphi (r_1 ) = +1 \text { and } r_1 \leftarrow r_2 \text { or }\\ &{} \varphi (r_1 ) = -1 \text { and } r_1 \rightarrow r_2. \\ \end{array}\right. } \end{aligned}$$

Suppose the data used to construct  are free of repetitive sequences and contain no false-positive overlaps. In such perfect data,  is a directed interval graph. Since we have contracted edges corresponding to nested reads (i.e., intervals),  is in fact a proper interval graph or indifference graph [38]. In addition  is directed in a manner consistent with the ordering of the intervals. More precisely, there is an ordering $\prec$ of the vertices (long reads) that satisfies the *umbrella property* [39]: $r_1\prec r_2\prec r_3$ and $r_1r_3\in E$ ( ) implies $r_1r_2,r_2r_3\in E$( ). We can interpret $r_1 \prec r_2$ to mean that $r_1$ extends $r_2$ to the left, i.e., towards smaller coordinate values in the final assembly. A “normal interval representation” and a linear order $\prec$ of the reads can be computed in ${\mathcal {O}}(|{\mathcal {R}}|)$ time [40, 41] for proper interval graphs.

Due to the noise in the data, however, we have to expect that the original overlap graph only approximates a proper interval graph. On the other hand, we have already obtained an orientation of the edges that – in ideal data – would be consistent with interval order. We therefore consider necessary conditions for directed indifference graphs and set out to enforce them.

First, we observe that  should be acyclic. Orientability w.r.t. a signature $\theta$, does not guarantee acyclicity since  still may contain some spurious “back-linking” edges due to unrecognized repetitive elements. The obvious remedy is to remove a $\Omega$-minimal set of directed edges. This amounts to solving an feedback arc set problem, which is known to be NP-complete in both weighted and unweighted versions, see [42] for a recent overview. We therefore resort to a heuristic that makes use of our expectations on the structure of : In general we expect multiple overlaps of correctly placed reads, i.e., *r* is expected to have several incoming edges from its predecessors and several outgoing edges exclusively to a small set of succeeding reads. In contrast, we expect incorrect edges to appear largely in isolation. This suggests to adapt Khan’s topological sorting algorithm [43]. In its original version, it identifies a source *u*, i.e., a vertex with in-degree 0, appends it to the list *W* of ordered vertices, and then deletes all its out-edges. It stops with “fail” when no source can be found before the sorting is complete, i.e., *W* does not contain all vertices of the given graph, indicating that a cycle has been encountered. We modify this procedure in two ways:

First, if multiple sources are available in a given step, we always pick the one with largest total $\Omega$-weight of edges incoming from the sorted set *W*. As a consequence, incomparable paths in  are sorted contiguously, i.e., a new path is initiated only after the previous one cannot be continued any further. Note that keeping track of the total input weight from *W* does not alter the ${\mathcal {O}}(|V|+|E|)$ running time of the Kahn’s algorithm.

Second, we replace the “fail” state by a heuristic to find an “almost source” to continue the sorting. Denote by $N_+(W)$ the out-neighborhood of the set *W* that has been sorted so far and consider the set $K := N_+(W)\setminus W$ the not-yet-sorted out-neighbors of *W*. These are the natural candidates for the next source. For each $u\in K$ we distinguish incoming edges *xu* from $x\in W$, $x\in K$, and $x\in V\setminus (W\cup K)$ and consider two cases:

(1): There is a $u\in K$ without an in-edge *xu* from some other $x\in K$. Then we choose among all vertices of this type the vertex ${\hat{u}}$ with the largest total $\Omega$-weight incoming from *W* because ${\hat{u}}$ then overlaps with most of the previously sorted reads.
(2): If for each $u\in K$ there is an in-edge *xu* from some other $x\in K$, then the candidate set *K* forms a strongly connected digraph. In this case we choose the candidate ${\hat{u}}\in K$ with the largest difference of $\Omega$-weights incoming from *W* and *K*, i.e., $\hat{u}:=\text{arg\,max}_{u\in K}\sum _{w\in W}\Omega (w,u) - \sum _{k\in K\setminus \{u\}}\Omega (k,u)$.

In either case, we add the edges incoming from $V\setminus W$ into ${\hat{u}}$ to the set *F* of edges that violate the topological order. It is clear from the construction that (i) *F* remains empty if  is a DAG since in this case a source is available in each step, and (ii) the graph  obtained by from  by deleting the edges in *F* is acylic since all in-edges to *u* in  emanate from *W*, the set of vertices sorted before *u*, and all out-edges from *u* point to the as yet unsorted set. Thus *F* is a feedback arc set for .

#### Lemma 9

*The modified Kahn algorithm can be implemented to run in* $O(|E|+|V|\log |V|)$ *time*.

#### *Proof*

Our modified Kahn algorithm keeps the not-yet-sorted vertices in a priority queue instead of a simple queue. The priority of a vertex $u\in V\setminus W$ depends on the number of total $\Omega$-scores of the in-edges *wu* with $w\in W\cap N^-(u)|$, $w\in K\cap N^-(u)$, and $w\in N^{-}(u)\cap V\setminus (W\cup K)$ respectively. Every time a vertex *v* is added to *W*, these values have to be updated for the out-neighbors $u\in N^+(v)$. Each update only comprises of the addition or subtraction of $\Omega (v,u)$ and the decision whether the second and/or third values are zero, and thus require total time $O(E(\mathbf {G}))$. Highest priority is given to vertices *u* with $N^-(u)\subseteq W$, i.e., true sources, next vertices $u\in K$ with $N^-(u)\cap K=\emptyset$, and the last tier is formed by the remaining vertices. Assuming an efficient implementation of the priority queue as a heap, the total effort for its maintenance is *O*(*E*) plus $O(|V|\log |V|)$ for the dequeuing operations, see e.g. [44, 45]. $\square$

It is possible that  is not connected. In this case, each connected component can be processed independently in subsequent processing steps. If the feedback set *F* is disjoint from $T_G$, then $T_G$ is still a $\Omega$-maximal spanning tree of . Otherwise, edges in $F\cap T_G$ need to be replaced. Lemma 5 that the replacement edges have to be drawn from non-tree edges between the vertex sets spanned by the connected components of $T_G\setminus F$. In principle, this can be done efficiently with specialized data structures for dynamic connectivity queries, in particular if $F\cap T_G$ is small [46]. However, the effort to run Kruskal’s algorithm again on  is by no means prohibitive, since the update has to be done only once.

### Golden paths

For perfect data, the directed proper interval graph  has a single source and a single sink vertex, corresponding to the left-most and right-most long reads $r'$ and $r''$, respectively. Furthermore, every directed path connecting $r'$ and $r''$ is a *golden path*, that is, a sequence of overlapping intervals that covers the entire chromosome. Even more stringently, every read $r\ne r',r''$ has at least one predecessor and at least one successor in . An undirected graph is a *proper interval graph* if there is a set of intervals, corresponding to the vertices, such that (i) no interval is properly contained within another, and (ii) two vertices are adjacent iff their intervals intersect. For perfect data, therefore, the overlap graph is a proper interval graph.

#### Lemma 10

[47] *A connected proper interval graph has a unique vertex order (other than the reversal of the order).*

The vertex order of a connected proper interval graph is therefore completely determined by fixing the orientation of single edge. In our case, the orientation is fixed by $r^*$. We choose the ascending vertex order, i.e., $r_1\prec r_2$ for every directed edge $r_1r_2$. A proper interval graph with such an orientation of edges is a *directed proper interval graph.*

For perfect data, therefore,  is directed proper interval graph and thus it would suffice to compute the unique topological sorting of . For real-life data, however, we cannot expect that even the acyclic graph  is a directed proper interval graph. Ploidy in eukaryotes may constitute a valid exception to this assumption, as differences in chromosomes ideally also cause diverging structures. However, given the high error rate of long reads, low sequence variation can only be differentiated in very high coverage scenarios or with the help of known ancestral relationships [48]; both are explicitly not targeted by LazyB. In practice, ploidy is commonly reduced even when sufficient coverage is available but can be recovered via variant calling [49]. High accuracy short read assemblies originating from different alleles can be expected to match equally well to the same long reads given their low quality. Therefore, also ploidy variation will normally be merged to a single consensus. Accordingly, we did not detect any mayor duplication issues in the human, fly, or yeast.

Our aim now is to approximate the DAG  by a disjoint union of connected directed proper intervals graphs. To gain some intuition for this task, we first consider reductions of directed graphs that expose longest paths.

A transitive reduction  of some directed graph  is a subgraph of  with as few edges as possible such that two vertices *x* and *y* are connected by a directed path in  if and only if they are connected by a directed path in *H* [50, 51]. It is well-known that every directed acyclic graph has a unique transitive reduction [51, Thm. 1]. This property allows us to call an edge *e* of an acyclic digraph *redundant* if $e\notin E$( ). Unfortunately, computation of the transitive reduction requires ${\mathcal {O}}(|V|\,|E|)$ time in sparse graphs and ${\mathcal {O}}(|V|^{\omega })$, where $\omega \approx 2.3729$ is the matrix multiplication constant. This is impractical for our purposes.

As a simpler analog of transitive reduction, we define the *triangle reduction*  of *H* as the digraph obtained from  by removing all edges $uw\in E$ ( ) for which there is a vertex *v* with $uv,vw\in E$( ).

#### Lemma 11

*If* *is a connected directed proper interval graph then (i)* *is a path, and (ii)*  = .

#### *Proof*

By Lemma 10,  has a unique topological sorting, i.e., $\prec$ is a unique total order. Property (ii) now is an immediate consequence of the umbrella property, and (iii) follows from the fact the transitive reduction is a subgraph of the triangle reduction and preserves connectedness. $\square$

As an immediate consequence of Lemma 11 we observe that if  is a connected induced subgraph of a directed proper interval graph , then  is an induced path in the triangle reduction  of . Of course, Lemma 11 does not imply that the triangle reduction  is a path. It serves as motivation, however, to identify long-read contigs as maximal paths in the triangle reduction  of the directed acycling graph . Since the topological sorting along any such path is unique, it automatically identifies all redundant non-triangle edges along a path.

We note that it is not necessary to first compute the transitive or triangle reduction if one is only interested in the maximal paths.

#### Lemma 12

*Let* *be a directed acyclic graph with triangle reduction* *and transitive reduction* . *Then* *P* *is a maximal path in* *if and only if it is a also maximal path in* *or* .

#### *Proof*

Every maximal path in  connects a source with a sink, since otherwise it could be extended at one the the ends. Now suppose that a longest path *P* contains an edge $e=r'r''$ that this not contained in the transitive reduction. By definition, then there is a path $P_{r'r''}$ of length at least 2 from $r'$ and $r''$, and since *H* is acyclic, no vertex in $P_{r'r''}$ lies along the path *P*. Thus $P'$ obtained from *P* by replacing *e* with $P_{r'r''}$ is again a path, which is strictly longer then *P*, contradicting the assumption that *P* was maximal. Thus *P* is contained  and . Since  and  is a subgraph of  and *P* is maximal in , it is also maximal in  and . $\square$

We note, furthermore, that the modified Kahn algorithm described above has the useful side effect of producing long runs of consecutive vertices $r_i,r_{i+1},\dots r_{j-1},r_j$. These can be used to effectively reduce the graph  by removing all arcs connecting non-consecutive vertices with any such run.

**Fig. 7:**
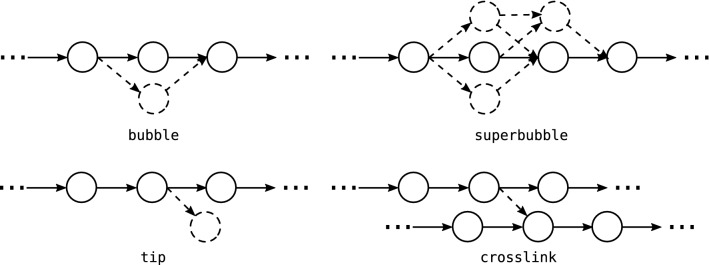
Examples of assembly graph defects in . Given two nodes , an $s-t$ path is a path starting in *s* and ending in *t*. A *simple bubble* consists of two vertex disjoint $s-t$ paths. This construct can be extended to *super-bubbles*, defined as a set of $s-t$ paths, exactly including all nodes reachable from *s* without passing *t* and *vice versa*. Bubbles and superbubbles are primarily the result of unrecognized overlaps. *Tips* are “side branches” that do not reconnect with the dominating paths and thus have distinct end-points. *Crosslinks*, finally, are connecting edges between two golden paths

The longest path terminating in a given vertex *x* can be computed with ${\mathcal {O}}(|E|)$ effort [52], suggesting that the explicit computation of reductions will not be helpful in practice. It also does not address the issue that the triangle-reduction  differs from a unique golden path by bubbles, tips, and crosslinks, see Fig. 7. Tips and bubbles predominantly are caused by edges that are missing e.g. due to mapping noise between reads that belong to a shared contig region. Remember that ploidy is collapsed to one haplotype due to the high error rates of long reads. Hence, any path through a bubble or superbubble yields essentially the same assembly of the affected region and thus can be chosen arbitrarily whereas tips may prematurely end a contig. Node-disjoint alternative paths within a (super-)bubble [53] start and end in the neighborhood of the original path. Tips either originate or end in neighborhood of the chosen path. As tips themselves may also be subject to mild noise, and crosslinks may occur near the start- or end-sites of the true paths, both are not always easily distinguished. Crosslinks represent connections between two proper contigs by spurious overlaps, caused, e.g., by repetitive elements that have escaped filtering. As crosslinks can occur at any position, a maximal path may not necessarily follow the correct connection and thus may introduce chimeras into the assembly.

We therefore have to expect that solving the longest path problem on  will sometimes follow spurious edges rather than locally more plausible choices since these may lead to overall shorter paths. As a remedy, we therefore aim to resolve the path choices based on local information. More precisely, we measure how well an edge *e* fits into a local region that forms an induced proper interval graph. Recall that a tournament is an orientation of a complete graph, and is called transitive if and only if it is acyclic [54].

#### Lemma 13

*If* *is a directed proper interval graph, then the subgraph* *induced by the closed outneighborhood* $N_+(r) := N^+(r)\cup \{r\}$ *is a transitive tournament*.

#### *Proof*

*By definition there is an arc from* *r* *to every* $u\in N^+(r)$. *Furthermore, we already know that* *has a unique topological ordering. The umbrella property therefore implies that there is an arc from* *u* *to* *v* *whenever* *u* *preceeds* *v* *in the unique topological ordering. Thus* *is a transitive tournament*. $\square$

For ideal data, the out-neighborhoods  form transitive tournaments, and their triangle reductions form induced subpath of . In fact, collecting results from the literature, it can be shown that is also necessary:

#### Theorem 14

*A connected directed graph* *H* *is a directed proper interval graph if and only if the out-neighorhood* $N^+(r)$ *is complete (and hence a transitive tournament) for every* $r\in V$ *and forms an interval in the (unique) vertex order*.

#### *Proof*

The equivalence of proper interval graphs and so-called closed graphs is shown in [55]. By definition, *H* is closed if it has so-called closed vertex ordering equivalent to the umbrella property [55]. Prop.2.2 in [56] states that a vertex ordering $\prec$ is closed if and only if the out-neighborhood is complete and forms an interval w.r.t. $\prec$. Together with the forward-orientation of the edges of *H* w.r.t. $\prec$, this in particular implies that $N^+(r)$ is transitive tournament. $\square$

An analogous result holds for the in-neighbors. Equivalently, proper interval graphs are also characterized by the fact that they admit a *straight* vertex order in which the in-neighbors of *r*, *r* itself, and then the out-neighbors of *r* appear consecutively [47].

For real data the subgraph  induced by the out-neighbors of *r* will in general violate the transitive tournament property. The problem of finding the maximum transitive tournament in an acyclic graph is NP-hard [57]. An approximation can be obtained, however, using the fact that a transitive tournament has a unique directed Hamiltonian path. Thus candidates for transitive tournaments in  can be retrieved efficiently as the maximal path $P_{rq}$ in  that connects *r* with an endpoint *q*, i.e., a vertex without an outgoing edge within . Using the topological order of , the maximal paths $P_{rq}$ can be traced back in ${\mathcal {O}}(|N_+(r)|)$ time for each endpoint $P_{rq}$.

For $P_{rq}$ we compute the number . The subgraph  with the largest value of $h_{rq}$ serves as approximation for the maximal transitive tournament with *r* as its top element. Its edge set  is used to define the *interval support* of an edge  as4Here, *d*(*r*, *e*) is the minimal number of edges in the unique path from *r* to *e* in the path formed by the edges in $H_r$. The interval support can be interpreted as the number of triangles that support *e* as lying within an induced proper interval graph. Importantly, it suffices to compute $\nu (e)$ for . The idea is now to choose, at every vertex *r* with more than one successor or precedssor in  the edges in $N^+(r)$ and $N^+(r)$ that have the maximal interval support. We observed empirically that determining the best path by greedily optimizing $\nu (e)$ at branch points results in contigs with a better solution quality compared to optimizing the weight $\Omega (e)$ of the spanning tree edges of $T_G$. Taken together, we arrive at the following heuristic to iteratively extract meaningful paths:

(i): Find a maximal path ${\mathbf {p}} = r_1,\ldots , r_n$ in  such that at every junction, we choose the incoming and outgoing edges *e* with maximal interval support $\nu (e)$.
(ii): Add the path ${\mathbf {p}}$ to the contig set if it is at least two nodes long and neither the in-neighborhood $N_-(r_1)$ nor the out-neighborhood $N_+(r_n)$ is already marked as “visited” in . Otherwise, we have found a tip if one of $N_-(r_1)$ or $N_+(r_n)$ was visited before and a bubble if both were visited. Such paths are assumed to have arisen from more complex crosslinks and can be added to the contig set if they exceed a user-defined minimum length.
(iii): The path ${\mathbf {p}}$ is marked “visited” in  and all corresponding nodes and edges are deleted from .
(iv): The procedure terminates when  is empty.

As the result, we obtain a set of paths, each defining a contig.

## Post processing of the path decomposition

### Consensus sequence

The final step is the retrieval of a consensus sequence for each path ${\mathbf {p}}$. This step is more complicated than usual due to the nature of our initial mappings. While we enforce compatible sets of unitigs for each pair of long reads, a shared unitig between edges does not necessarily imply the same genomic coordinate. We have to consider four distinct situations: (i) Unitigs can be long enough that we gain triples $r_i, r_{i+1}, r_{i+2} \in V({\mathbf {p}})$ such that an $s \in {\mathscr {S}}_{r_ir_{i+1}} \cap {\mathscr {S}}_{r_{i+1}r_{i+2}}$ exists but $r_i$ and $r_{i+2}$ share no interval on *s*. Such triples can occur chained. (ii) Unitigs of genomic repeats may remain in the data. Such unitigs may introduce pairwise distinct edges $e_i,e_j,e_k$ that appear in this order, denoted by $e_i \prec e_j \prec e_k$, along the path ${\mathbf {p}}$ such that $s\in {\mathscr {S}}_{e_i}$ and $s\in {\mathscr {S}}_{e_k}$ but $s \notin {\mathscr {S}}_{e_j}$, therefore creating disconnected occurrences of *s*. (iii) Similarly, proximal repeats may cause inversions in the order of two unitigs $s, s' \in {\mathscr {S}}_{e_i} \cap {\mathscr {S}}_{e_k}$, w.l.o.g $e_i \prec e_k$. This scenario cannot appear on neighboring edges, as the shared node has a unique order of *s* and $s'$. Hence, either *s* or $s'$ must be missing in an intermediary edge $e_l$ due to the consistency constraints in the original graph, resulting in a situation as described in (ii). (iv) Finally, true matches of unitigs may be missing for some long reads due to alignment noise, which may also yield a situation as in (ii).

To address (i), we collect all instances of a unitig in the path independent of its context. We create an undirected auxiliary graph $U_s$ with a vertex set $V(U_s):= \{e \in E({\mathbf {p}}) \mid s \in {\mathscr {S}}_{e}\}$. We add edges for all edge-pairs that share an overlap in *s*. Any clique in this graph then represents a set of edges that share a common interval in *s*. We assign each edge a unique cluster index $c_s^e$, according to a minimal size clique decomposition. As finding a set of maximal cliques is NP-hard, we instead resort to a $\mathcal {O}(|V|/(\log |V|)^2)$ heuristic [58]. We address (ii-iv) with the help of a second index $g_s^e$, where $g_s^{e_i} \ne g_s^{e_k}$ for two edges $e_i, e_k$ if and only if an edge $e_j$ exists such that $e_i \prec e_j \prec e_j$ and $s \notin {\mathscr {S}}_{e_j}$.

Finally, we can now create a multigraph *M* consisting of vertex triples $\{ (s, c_s^e, g_s^e) \mid s \in {{\mathscr {S}}_{e}} \text { with } e\in E({\mathbf {p}})\}$. We add edges $(s, c_s^e, g_s^e) \rightarrow (s', c_s'^e, g_s'^e)$ if and only if $s \prec s'$ on an edge *e* and no element $s''$ exists such that $s \prec s'' \prec s'$. The resulting graph *M* is cycle free and thus uniquely defines the positions of all unitigs. Nodes represent the sequence of the common interval on the unitig *s* as attributed to the clique $c_s^e$. Edges represent the respective sequence of long reads between *s* and $s'$, or a negative offset value if unitigs overlap. We take an arbitrary node in *M* and set its interval as the reference point. Positions of all other nodes are progressively built up following a topological order in this graph. If multiple edges exist between two nodes in this process an arbitrary but fixed edge is chosen to estimate the distance between nodes.

At this point, all sequence features are embedded in the same coordinate system. The reference contig is obtained as an in principle arbitrary projection of the read sequences. In practice, the short-read unitigs are used wherever available because of their much higher sequence quality. At the same time, we can map the features of each long read to their respective position in this newly constructed reference. This information can be directly fed into consensus based error correction systems such as racon [59].

## Benchmarking

To demonstrate the feasibility of our assembly strategy we applied LazyB to data sets from previously published benchmarks of Nanopore assemblies. For yeast (*S. cerevisiae*) we used Nanopore sets ERR1883389 for lower coverage, ERR1883399 for higher coverage, and short-reads set ERR1938683, all from bioproject PRJEB19900 [60]. For comparison we used the reference genome R64.2.1 of strain S288C from the SGD. For fruit fly (*D. melanogaster*) we used the Oxford Nanopore and Illumina raw data of bioproject PRJNA433573 [61], and the FlyBase reference genome 6.30 (http://www.flybase.org). For Human we uses accession SRX6356866-8 of bioproject PRJNA549351 [62] for long reads and SRA292482 [63] for short reads. Assemblies are compared against the NCBI reference genome GRCh38.p13.

Sequencing data were downsampled to approximately 5$\times$ and 10$\times$ nanopore coverage for long reads, respectively, and Illumina coverage sufficient for short-read anchors. We compare results to the most widespread competing assembler Canu [10] and the faster Wtdbg2 [13], both demonstrating the disadvantages of long-read-only strategies especially in low coverage scenarios, although Wtdbg2 requires considerately less coverage in comparison. Additionally, we benchmarked two tools implementing the most closely related concept: DBG2OLC [23] and most recent competitor HASLR [21]. Finally, we added Wengan [19] as a leading but conceptually very unique alternative. Wengan uses long reads to scaffold a short read assembly and therefore exhibits very high levels of completeness even when presented with very few reads. It defaults to the pre-existing contigs or statistically insignificant scaffolds when nothing else can be done. This behavior can be mimicked partially with LazyB by merging our assembly to the same short-read-only assembly as used by Wengan. However, we strictly limit merging to regions with strong support from long read contigs to avoid spurious scaffolds. While not ideal, we used the pre-existing tool Quickmerge [64] to investigate such effects. At very low long read coverage, integration with a short read assembly is generally advisable to close gaps in long reads unavoidably arising on complex genomes. For reference, we also provide the statistics for short-read only assemblies created with ABySS [29] on the same sets of reads used to create the “anchors” to show the advantage of hybrid assembly even at a low coverage of long reads. The same short read assembly was used also for Wengan and Quickmerge on LazyB. Quality was assessed via alignment to a reference genome by the QUAST tool [65].

Table 3 summarizes the benchmarking results. Unsurprisingly, LazyB produced consistently better results than Canu and Wtdbg2, increasing genomic coverage at a lower contig count. Due to our inclusion of accurate short-read unitigs, overall error counts are also significantly lower than on Canu. Most notably, Canu was unable to properly operate at the 5$\times$ mark for both data sets. Only insignificant portions of the yeast genome could be assembled, accounting for less than 15% of the genome. Canu completely failed for fruit fly, even after adapting settings to low coverage. Wtdbg2 performed only marginally better, although it managed to assemble 6% of fruit fly at low coverage. Even at 5$\times$, LazyB already significantly reduces the number of contigs compared to the respective short-read assemblies, while retaining a reasonably close percentage of genome coverage. At only 10$\times$ coverage for fruit fly, we were able to reduce the contig count 10-fold at better error rates. For human, LazyB manages at 39-fold decrease of the number contigs, albeit at a loss of greater 10% coverage. This difference appears to be a consequence of the high fragmentation of unitigs in the abundant repeat regions of the genome, rendering them too unreliable as anchors. Results are indeed in line with unitig coverage. While HASLR produced the fewest mis-assemblies, it creates significantly more and shorter contigs that cover a much smaller fraction of the genome. As a consequence it has the least favorable NA50 values of all tools. For fruit fly at 10$\times$, it results in four times as many contigs and covers 10% less of the genome, with a 12 times lower NA50. While an improvement to Canu, it also struggles on datasets with low Nanopore coverage. DBG2OLC shows great promise compared to our own method, but similarly fails to operate well on very low coverage datasets. For yeast at 5$\times$, less then 50% the genome can be reconstructed. In fruit fly even less then 25% can be assembled at about 2 times the error rate of LazyB. At 10$\times$, DBG2OLC reconstruct a similar proportion of the genome, albeit at high error rates. While it produces about 100 fewer contigs for fruit fly, this achievement is offset by over 350 (4.7 times more) mis-assemblies.

**Table 3:** Assessment of assembly qualities for LazyB, Canu Wtdbg2, HASLR, Wengan and short-read only assemblies for two model organisms

Org.	X	Tool	Compl. [%]	#ctg	#MA	MM	InDels	NA50
Yeast	${\sim} 5\times$	LazyB	90.466	127	9	192.56	274.62	118843
		LazyB+QM	94.378	64	12	174.77	245.05	311094
		Canu	14.245	115	5	361.47	2039.15	–
		Wtdbg2	22.237	177	0	849.07	805.31	–
		HASLR	64.158	111	1	14.87	34.86	60316
		DBG2OLC	45.645	53	20	2066.64	1655.92	–
		Wengan	95.718	41	11	49.14	68.47	438928
	${\sim} 11\times$	LazyB	97.632	33	15	193.73	300.20	505126
		LazyB+QM	94.211	34	14	234.59	329.4	453273
		Canu	92.615	66	15	107.00	1343.37	247477
		Wtdbg2	94.444	42	8	420.96	1895.28	389196
		HASLR	92.480	57	1	7.89	33.91	251119
		DBG2OLC	97.689	38	25	55.06	1020.48	506907
		Wengan	96.036	37	4	32.35	53.04	496058
	${\sim} 80\times$	Abyss	95.247	283	0	9.13	1.90	90927
Fruit fly	${\sim} 5\times$	LazyB	71.624	1879	68	446.19	492.43	64415
		LazyB+QM	75.768	1164	79	322.49	349.29	167975
		Canu	–	–	–	–	–	–
		Wtdbg2	6.351	2293	2	916.77	588.19	–
		HASLR	24.484	1407	10	31.07	58.96	–
		DBG2OLC	25.262	974	141	1862.85	969.26	–
		Wengan	81.02	2129	192	105.35	123.33	77215
	${\sim} 10\times$	LazyB	80.111	596	99	433.37	486.28	454664
		LazyB+QM	80.036	547	100	416.34	467.14	485509
		Canu	49.262	1411	275	494.66	1691.11	–
		Wtdbg2	41.82	1277	155	2225.12	1874.01	–
		HASLR	67.059	2463	45	43.83	84.89	36979
		DBG2OLC	82.52	487	468	739.47	1536.32	498732
		Wengan	84.129	926	237	114.96	154.03	221730
	${\sim} 45\times$	Abyss	83.628	5811	123	6.20	8.31	67970
Human	${\sim} 10\times$	LazyB	67.108	13210	2915	1177.59	1112.84	168170
	${\sim} 43\times$	Unitig	69.422	4146090	252	93.07	13.65	338
	${\sim} 43\times$	Abyss	84.180	510315	2669	98.53	25.03	7963

LazyB outperforms Canu and Wtdbg2 in all categories, while significantly reducing contig counts compared to short-read only assemblies. While HASLR is more accurate, it covers significantly lower fractions of genomes at a higher contig count and drastically lower NA50. DBG2OL produces few contigs at a high NA50 for higher coverage cases, but calls significantly more mis-assemblies. Wengan performs well for yeast, but produces more misassemblies at a higher contig count on fruit fly. Merging LazyB assemblies to the set of short read contigs (+QM) has a positive effect at 5$\times$ long-read coverage but negligible influence at higher coverage. Mismatches and InDels are given per 100 kb. Accordingly, errors in LazyB ’s unpolished output constitute $<1$% except for human. Wtdbg2 assemblies were not polished. Column descriptions: X coverage of sequencing data, *compl*eteness of the assembly. #ctg: number of contigs, #MA: number of mis-assemblies (breakpoints relative to the reference assembly) M is Matches and InDels relative to the reference genomes. NA50 of correctly assembled contigs. We follow the definition of QUAST: Given a set of fragments as the sub-regions of the original contigs that were correctly aligned to the reference, the NA50 (also named NGA50) is defined as the minimal length of a fragment needed to cover 50% of the genome. This value is omitted when $< 50\%$ is correctly recalled

A Comparison with Wengan is more complex due to its unique method. Integration of the *AByss* short-read assembly has little effect on LazyB at 10$\times$, as the genomes of both yeast and fruit fly are already well covered. At 5$\times$, contigs are around halved with negligible adverse consequence to misassemblies, furthering our advantage. Since the merging step cannot significantly increase genome coverage, we did not consider it for other tools. On yeast, Wengan improves LazyB results marginally both at 5$\times$ and 10$\times$. On fruit fly, in turn, LazyB produced substantially better assemblies. At 5$\times$, 250 fewer contigs (11%) were created at nearly 3 times fewer misassemblies, although 10% less of the genome is covered. Integration of the short read assembly widens the gap to 965 (45%) fewer contigs and increases the fraction of covered reference by an additional 5%. At 10$\times$ LazyB calls over 1.5-times fewer contigs with less than half the number of misassemblies out of the box.

**Fig. 8:**
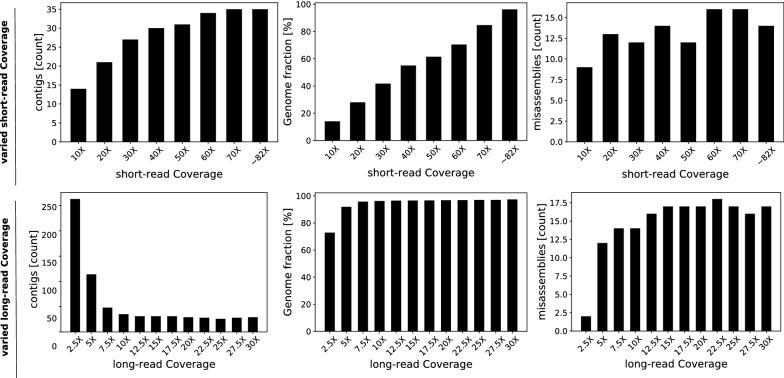
Variation of short (Top) and long read (Bottom) coverage for yeast. Long read coverage is set fixed at 10$\times$ or short read coverage at maximum ($\sim 82\times$) respectively. Result are given as the statistics of LazyB assembly : (Left) number of contigs, (Middle) fraction of the reference genome covered, and (Right) the number of misassemblies

**Fig. 9:**
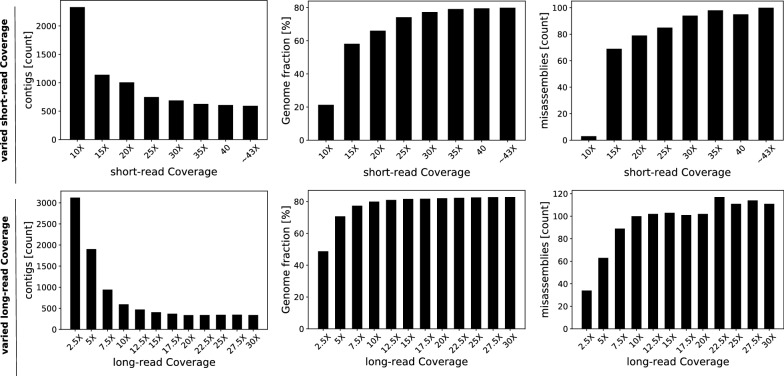
Variation of short-(Top) and long-read (Bottom) coverage for fruit fly. Long read coverage is set fixed at 10$\times$ or short read coverage at maximum ($\sim 43\times$) respectively. Result are given as the statistics of LazyB assembly: (Left) number of contigs, (Middle) fraction of the reference genome covered, and (Right) the number of misassemblies

In order to establish the limits of suitable coverage for our method, we set up two simple range tests: coverage of either long reads and short reads is systematically varied while the other remains fixed; see Figs. 8 and 9. Unsurprisingly, the quality of LazyB assemblies increases with coverage, for both short and long reads. Short read coverage is positively correlated to assembly quality with only some notable saturation in fruit fly. Conversely, long read coverage reaches its optimum at 10$\times$ in both organism. While no notable improvements can be achieved after this point, also no negative trend can be seen in the tested range up to 30$\times$. At 5$\times$ long reads the number of contigs increases, but genome coverage remains nearly stable. Only at 2.5*x* also a notable drop in coverage. The quality of the assembly remains respectable even then, however.

**Table 4:** Assessment of assembly qualities for very low coverage of long reads at maximum short read coverage ($\sim 43\times$) on human

X	Tool	Compl.[%]	#ctg	#MA	MM	InDels
1$\times$	LazyB	10.329	38342	109	831.14	773.86
	LazyB+QM	19.023	39588	280	418.26	332.60
	Wtdbg2	0.285	6724	21	1477.08	313.01
	DBG2OLC	0.771	9475	10	1050.82	202.51
	Wengan	67.220	116201	4057	208.38	142.51
2$\times$	LazyB	25.865	69043	432	938.26	814.44
	LazyB+QM	35.151	68180	693	648.35	521.57
	Wtdbg2	2.069	24346	126	1334.25	384.36
	DBG2OLC	3.904	34069	58	959.98	492.63
	Wengan	72.915	90784	4954	259.42	195.63
2.5$\times$	LazyB	32.126	70690	692	978.28	825.80
	LazyB+QM	39.796	69053	917	753.19	606.78
	Wtdbg2	3.702	32031	163	1202.70	412.21
	DBG2OLC	7.104	42864	170	1044.31	679.40
	Wengan	74.835	80605	5115	271.25	211.36
${\sim} 43\times$	ABySS	84.180	510315	2669	98.53	25.03

Column descriptions: X: coverage of sequencing data, *compl*completeness of the assembly. #ctg: number of contigs, #MA: number of mis-assemblies (breakpoints relative to the reference assembly) M isMatches and InDels: relative to the reference genomes

**Fig. 10:**
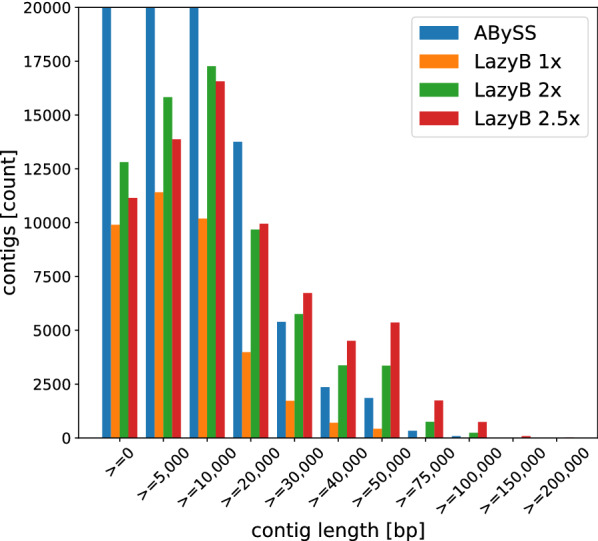
Length distribution of contigs for the human short read assembly of ABySS at 43$\times$ contrasted to LazyB assemblies at 1$\times$, 2$\times$ and 2.5$\times$ coverage. Counts for ABySS at low contig lengths have been cut off to allow better visibility of the desired region. LazyB surpasses total counts

We chose human for further testing of this threshold since it is the largest and most complex genome with a high quality reference for which suitable Oxford Nanopore data were available to us. In line with our previous tests, we consider even lower long read coverage than before (1–2.5$\times$; see Table 4). Wtdbg2 and DBG2OLC failed to assemble significant regions of the genome. Canu had to be excluded as the pipeline failed completely. Wengan’s results appear impressive at first glance, calling up to nearly 75% of the genome correctly, at over 6 times fewer contigs than the short-read assembly.

However, the mystery behind this result is hidden in the vastly increased number of misassemblies, close to doubling the already high misassembly rate of the underlying short-read assembly. While LazyB produces shorter contigs and covers much less of the genome (up to 32%) in comparison, it does so at an error rate proportional to or better than the short read assembly. At this level of coverage, it seems unlikely to recover the genome both completely and correctly, but rather, a trade-off between both occurs. Unfortunately, we were not able to adjust settings on either tool to match the behavior of the other for a direct comparison. Yet, we can conclude that LazyB is well suited to improve the contiguity of short reads assemblies *ad hoc*. LazyB produces significantly larger contigs at 2$\times$ and 2.5$\times$. Total counts of large contigs increase despite covering significantly less of the genome (84% vs 25-32%); see Fig. 10. Merging short and long read assembly with Quickmerge improves recall and reduces the number of contigs (except for 1$\times$) at the cost of a commensurate increase of misassemblies.

**Fig. 11:**
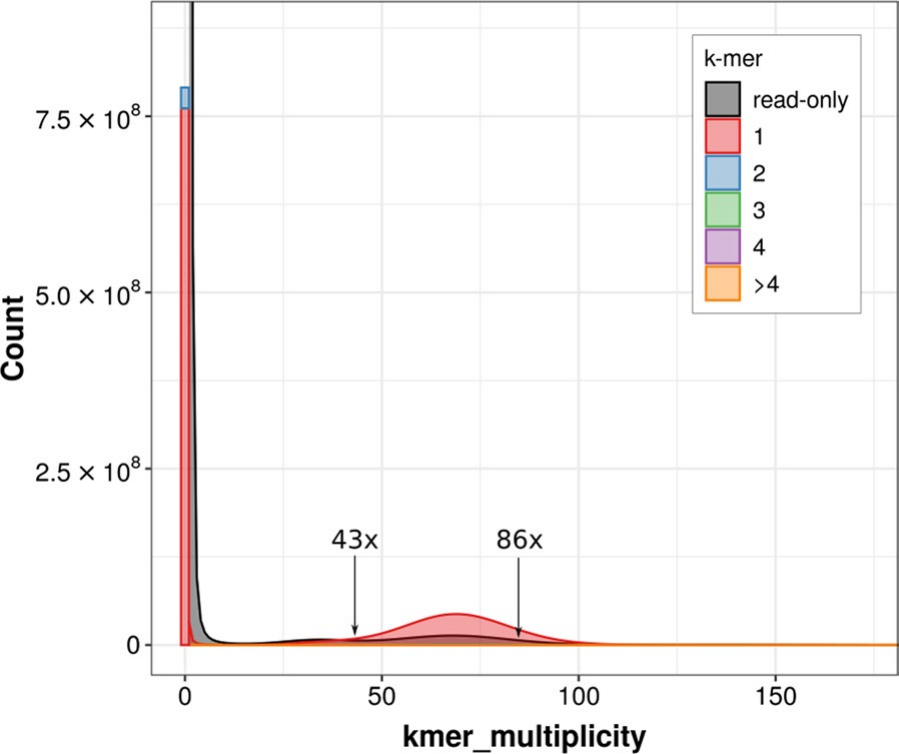
Copy number spectrum plot generated by Merqury as *k*-mers ($k=21$ as recommended) plotted as stacked histograms colored by the copy numbers found in the 10$\times$ long-read coverage assembly of LazyB. The typical peak generated at slightly less than twice the short-read coverage ($2\cdot 43\times = 86\times$) in concordance with the absence of higher copy numbers clearly indicate the presence of only a single mixed haplotype. The small elevation of *k*-mers only found in reads at the level short-read coverage can be attributed to few haplotype regions not fitting well to the mixture. The slight shift in short-read coverage versus *k*-mers arises out of the uncorrected high error rate of long reads

To address our assertion that ploidy is effectively reduced to a mixed haplotype by our method, we can follow two general strategies for verification. Given a reference, the presence of separated haplotypes will appear as duplicate overlapping alignments against the reference. None of the QUAST statistics gathered for LazyB show duplication beyond 2%. As a secondary, reference independent, method, copy number spectrum plots can be used. Multiplicty of *k*-mers of short read is gather and colored by the number of times it is found in a given assembly. In diploid assemblies, we would expect two peaks: at 2 times base coverage for shared stretches of the genome and at base coverage for unique regions. Analysis with Merqury [66] revealed only a single peak (Fig. 11) at twice the coverage for the most complex assembly on human, thus indicating a mixed haplotype as predicted.

**Table 5:** Assessment of running times for all tools. Resource consumptions for LazyB are shown for the complete assembly process comprising (1) ABySS unitig assembly; (2) Mapping of unitigs to long reads and (3) running LazyB itself, denoted by A+m+ LazyB, and the the last step only, denoted by LazyB

$\times$	Tool	Time	RAM (MB)
Yeast			
	ABySS unitig	00:00:11:03	2283
	ABySS full	00:00:20:01	2283
${\sim} 5\times$	Mapping	00:00:00:05	540
	LazyB	00:00:00:30	136
	A+m+ LazyB	00:00:11:38	2283
	Canu	00:10:23:55	2617
	Wtdbg2	00:00:13:08	698
	HASLR	00:00:06:44	4922
	DBG2OL	00:00:31:46	1141
	Wengan	00:00:06:45	4400
	A+Wengan	00:00:26:46	4400
${\sim} 11\times$	Mapping	00:00:00:15	1544
	LazyB	00:00:01:46	362
	A+m+ LazyB	00:00:13:04	2283
	Canu	00:13:44:16	6779
	Wtdbg2	00:00:29:28	1142
	HASLR	00:00:08:09	4922
	DBG2OL	00:00:51:13	1264
	Wengan	00:00:14:29	4421
	A+Wengan	00:00:34:30	4421
Fruit fly			
	ABySS untig	00:02:32:39	25344
	ABySS full	00:04:56:03	25346
${\sim} 5\times$	Mapping	00:00:02:43	6433
	LazyB	00:00:08:33	613
	A+m+ LazyB	00:02:43:55	25344
	Canu	02:13:51:39	7531
	Wtdbg2	00:01:37:41	3395
	HASLR	00:01:30:33	5531
	DBG2OL	00:07:58:22	6151
	Wengan	00:01:41:26	5394
	A+Wengan	00:06:10:29	25346
${\sim} 10\times$	Mapping	00:00:06:11	9491
	LazyB	00:00:11:57	2241
	A+m+ LazyB	00:02:50:47	25344
	Canu	07:04:08:28	7541
	Wtdbg2	00:04:02:43	5024
	HASLR	00:01:43:21	5553
	DBG2OL	02:07:32:01	17171
	Wengan	00:02:28:51	5323
	A+Wengan	00:07:24:54	25346

Step (1) is often not needed as short-read assemblies are available for many organisms

Similarly, Wengan requires a full ABySS assembly as a its basis. Resources are only compared for yeast and fruit fly, because Canu cannot be run for human in acceptable time and resource-constraints on our equipment. As all tools except LazyB and DBG2OL are parallelized, running times are given as the sum of time spent by all CPUs. Therefore, computational effort is measured rather than wallclock time. ABySS greatly dominates the LazyB pipeline and to a lesser degree also Wengan. Nevertheless, LazyB is faster by a factor of $>60$ compared to Canu, $\approx 3$ compared to DBG2OL, and $\approx 2.5$ to Wengan

The resource footprint of LazyB is small enough to run on an off-the-shelf desktop machine or even a laptop. The total effort is, in fact, dominated by the computation of the initial unitig set from the short reads. We expect that an optimized re-implementation of LazyB will render its resource consumption negligible. Compared to the competing Canu assembler, the combination of ABySS and the python-prototype of LazyB is already more than a factor of 60 faster. In terms of memory, given precomputed unitigs LazyB also requires $3-18$ times less RAM than Canu, see Table 5. LazyB is also significantly faster than the more resource efficient Wtdbg2 and Wengan. Most notably, we were able to assemble the human genome within only 3 days, while Canu could not be run within our resource constraints. HASLR shows a similar distribution of running times between tasks, overall operating slightly faster. We could not process our human test set with HASLR, however. A human DBG2OLC assembly can be estimated to take several weeks without manual parallelization for a single set of parameters, with authors recommending several possible alternatives for optimization. We therefore include only the results for LazyB here, and leave a more detailed comparison of the performance for very complex genomes for a proper follow-up experiment.

## Discussion and outlook

We demonstrated here the feasibility of a new strategy for sequence assembly with low coverage long-read data. Already the non-optimized prototype LazyB, written entirely in python, not only provides a significant improvement of the assembly but also requires much less time and memory than state-of-the-art tools. This is achieved by avoiding both a correction of long reads and an all-*vs*-all comparison of the long reads. Instead, we use rigorously filtered short-read unitigs as anchors, sparsifying the complexity of full string-graph construction. LazyB then uses a series of fast algorithms to consistently orient this sparse overlap graph, reduce it to a DAG, and sort it topologically, before extracting contigs as maximum weight paths. This workflow relies on enforcing properties of ideal overlap graphs that have not been exploited in this manner in competing sequence assembly methods.

The prototype implementation leaves several avenues for improvements. We have not attempted here to *polish* the sequence but only to provide a common coordinate system defined on the long reads into which the short-reads unitigs are unambiguously embedded to yield high-quality parts of the LazyB -assembly. The remaining intervals are determined solely by long-read data with their high error rate. Multiple edges in the multigraph constructed in the assembly step correspond to the same genome sequence, hence the corresponding fragments of reads can be aligned. This is also true for alternative paths between two nodes. This defines a collection of alignments distributed over the contig, similar to the situation in common polishing strategies based on the mapping of (more) short-read data or long reads to a preliminary assembly. Preliminary tests with off-the-shelf tools such as racon [59], however, indeed improve sequence identity but also tend to introduce new translocation breakpoints. We suspect this is the consequence of InDels being much more abundant than mismatches in Nanopore data, which is at odds with the Needleman–Wunsch alignments used by polishing tools.

We suspect that further improvements can be achieved by improving the quality of the initial overlap graph. Conceivably, more stringent filtering of the short-read unitigs against multi-copy sequences with similarities comparable to the expected error levels in the long reads can reduce spurious edges. It may also be worthwhile to compute pairwise alignments of the long-read sequences that form edges in overlap graph to confirm overlapping intervals. However, as we have seen, classical aligners do not perform satisfactorily, presumably due to the InDel-dominated error profile of the current long read sequencing methods. Better alignment approaches would also be required in the finishing steps. It remains to be seen whether dedicated aligners methods, such as the current-level modeling approach of QAlign [67] are able to resolve these issues.

A prominent category of mis-assemblies within the LazyB contigs are inherited from chimeric reads. This therefore suggests an iterative approach: Subsampling the long-read set will produce more fragmented contigs, but statistically remove chimeric reads from the majority of replicate assemblies. Final contigs are constructed in a secondary assembly step by joining intermediary results. It might appear logical to simply run LazyB again to obtain a “consensus” assembly, where intermediary contigs play the role of longer reads with mapped anchors. In preliminary tests, however, we observed that this results in defects that depend on the sampling rate. The question of how to properly design the majority calling to construct a consensus assembly remains yet to be answered.

Finally, a proper pipeline needs to be established to join short-read assemblies and very low coverage LazyB assemblies. While Quickmerge appears to produce satisfying results (and short-read contigs in regions not covered by the long-read assembly could be fished out as the set of uninvolved contigs in this process), we presume a dedicated method may yield even better results.

## Data Availability

The reported data can be accessed at http://tunicatadvexillum.bioinf.uni-leipzig.de/Home.html.

## Abbreviations

GO: Gene ontology
miRNA: MicroRNA
ML: Maximum likelihood
ncRNA: Non-coding RNA
